# Myocardial Damage in a Highly Suspected Case With Paraneoplastic Pemphigus: A Case Report and Literature Review

**DOI:** 10.3389/fmed.2022.917050

**Published:** 2022-06-13

**Authors:** Xiao Du, Miao Zhang, Shilan Zhang, Feng Tian, Tie Wen, Ling Liu

**Affiliations:** ^1^Department of Cardiovascular Medicine, The Second Xiangya Hospital, Central South University, Changsha, China; ^2^Research Institute of Blood Lipid and Atherosclerosis, Central South University, Changsha, China; ^3^Modern Cardiovascular Disease Clinical Technology Research Center of Hunan Province, Changsha, China; ^4^Cardiovascular Disease Research Center of Hunan Province, Changsha, China; ^5^Department of Emergency Medicine, Second Xiangya Hospital, Central South University, Changsha, China; ^6^Emergency Medicine and Difficult Diseases Institute, Second Xiangya Hospital, Central South University, Changsha, China

**Keywords:** paraneoplastic pemphigus, myocardial damage, tumor, autoimmune disease, autoantibodies

## Abstract

Paraneoplastic pemphigus (PNP) is a rare mucocutaneous autoimmune disease. It has multiple clinical accompanied symptoms by affecting various types of epithelia, including the gastrointestinal and respiratory tract. However, an extensive review of the literature found no cases of PNP associated with myocardial damage. Here, we present a 56-year-old male patient with clinically and histopathologically typical paraneoplastic pemphigus (PNP), who had sustained myocardial injury due to non-cardiac disease involvement. Therefore, we suppose that, when persistent cardiac necrosis markers are elevated in patients with paraneoplastic pemphigus (PNP), the possibility of concomitant myocardial damage should get more attention from clinicians to obtain quick diagnosis and treatment.

## Introduction

Paraneoplastic pemphigus (PNP) is a rare mucocutaneous autoimmune disease that is characterized by painful mucosal erosions, ulcerations, various forms of skin lesions, and is associated with the presence of an underlying neoplasm (1). About 500 cases have been reported in the literature since they were first described in 1990 (2). Stomatitis is the most characteristic feature of PNP, which usually is the first sign to appear and persists over the course of the disease (3). In addition to stomatitis, mucositis of the pharynx, larynx, and esophagus may occur (4). Moreover, anogenital involvement has been observed in PNP (5). In some cases, mucosal involvement is the only sign of PNP (6). Also, up to 92.8% of cases were reported to involve the respiratory epithelium of patients (7). However, the literature review did not identify any cases of PNP associated with myocardial damage. Here, we report a 56-year-old male patient with clinically and histopathologically typical PNP, who presented with persistent myocardial injury due to non-cardiac involvement.

## Case Presentation

**Figure 1:**
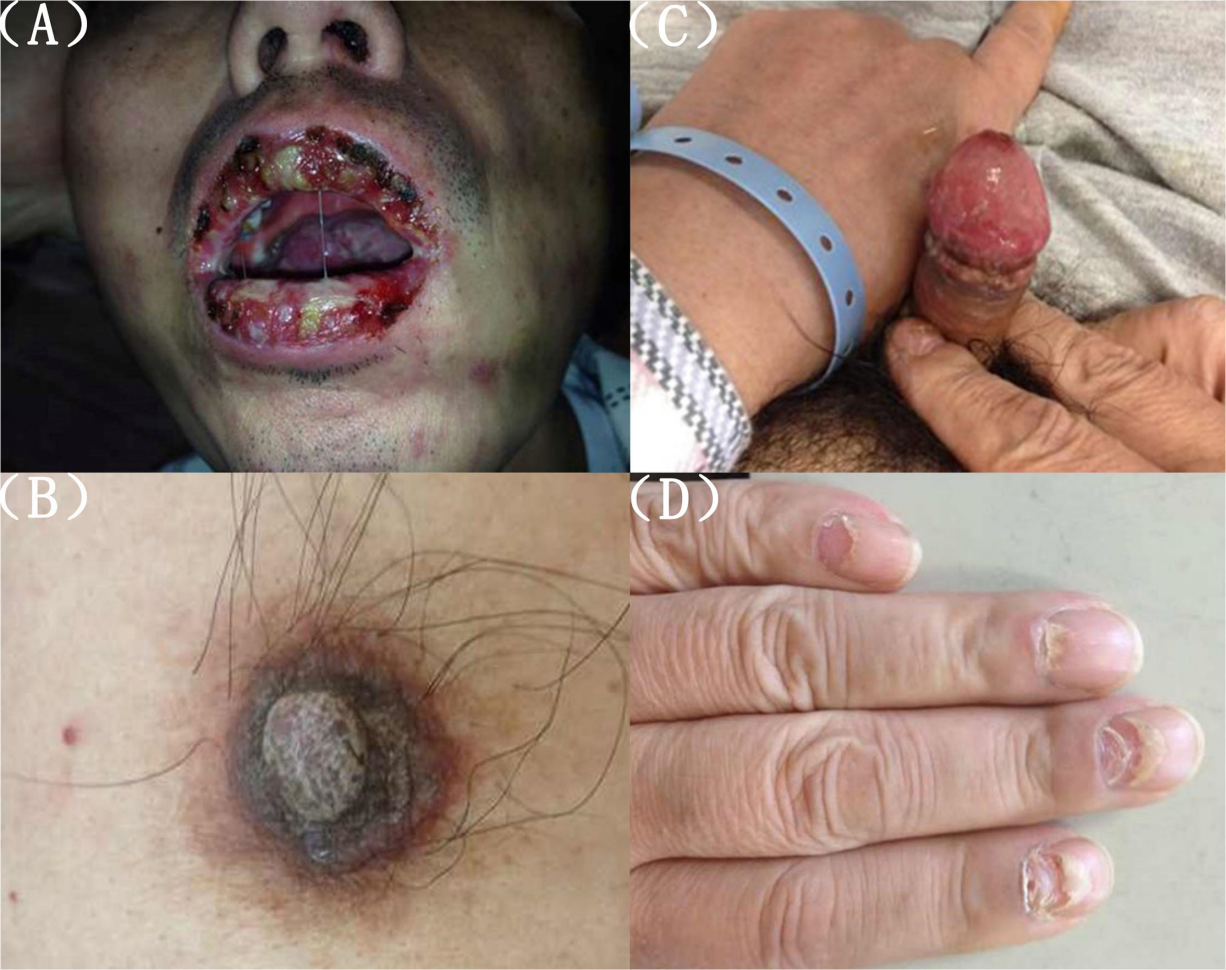
**(A)** Peri-oral lesions. Palatal and buccal mucosal ulcers and maculopapular lesions, lip crusts. **(B–D)** Scales on nipple, ulceration of genital, and nail lesions on fingers and toes.

**Figure 2:**
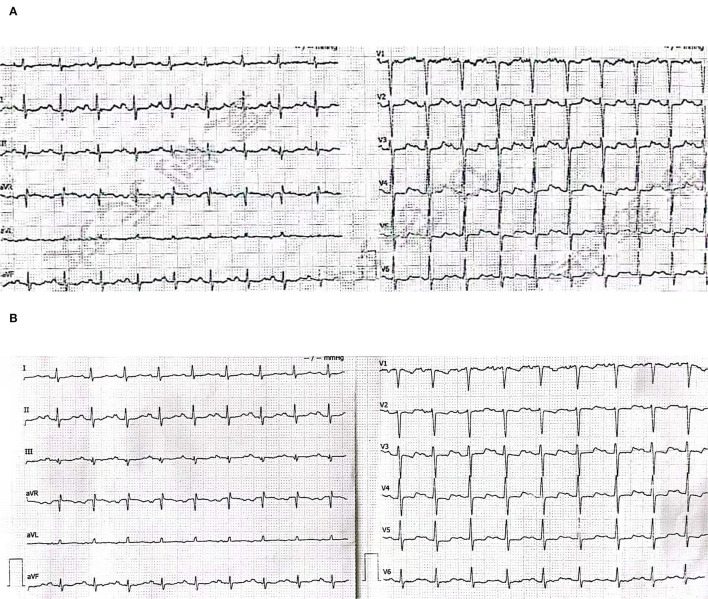
**(A)** Electrocardiogram for the first (emergency department). Sinus tachycardia, mild depression of the ST segment in V3~V5, T wave inversion in lead V1, and bidirectional in lead V4. **(B)** Electrocardiogram for the second time (the ward of the department of cardiovascular disease).

**Figure 3:**
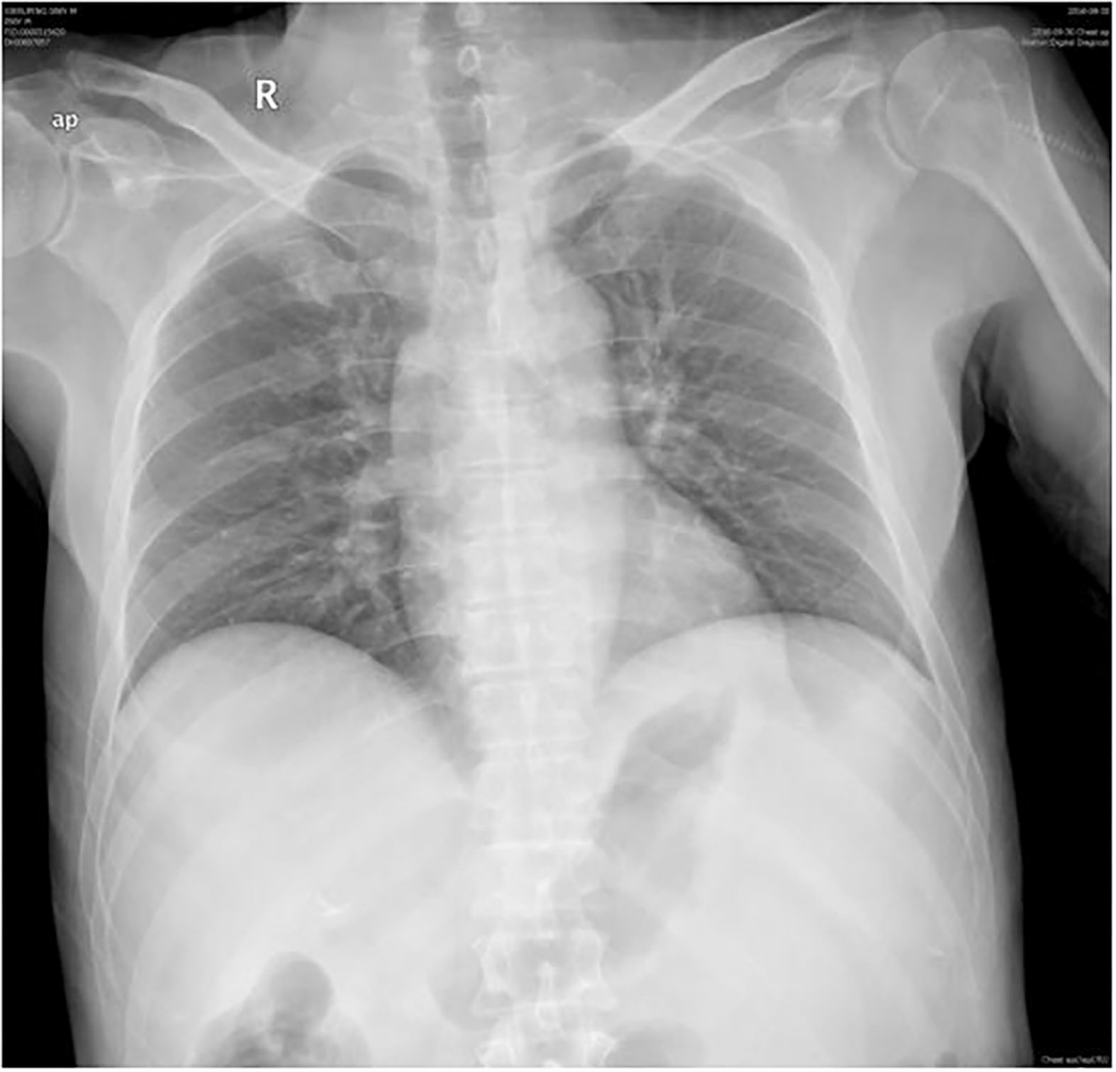
Chest radiography. The cardio-thoracic proportion of 0.47, elevating dexter diaphragm.

A 56-year-old male presented to the emergency department with 2 days of persistent dyspnea and chest pain. He had visited stomatology and dermatology departments several times in the last 2 months for painful oral mucosal erosions that have been diagnosed as “allergy,” “pemphigus,” “erythema multiforme,” and “oral lichen planus” in different hospitals. Metronidazole, cephalosporins, fluconazole, and dexamethasone were administered sequentially, but no significant improvement in symptoms was observed. He had no family history of hypertension, coronary heart disease, occupational exposure, or any autoimmune disease. On his physical examination, blood pressure and heart rate were 132/74 mmHg and 118 beats/min, respectively, and the respiratory rate was 21 breaths/min. He had ulcerated and bullous lesions on the palate, cheek mucosa, and blood crust on his lip (Figure 1A). A cardiovascular examination revealed regular heart sounds without murmurs. An electrocardiogram showed sinus tachycardia, mild depression of ST-segment in V_3_~V_5_, T wave inversion in lead V1, and bidirectional in lead V4 (Figure 2A). Chest radiography showed a cardio-thoracic proportion of 0.47, an elevation of the dexter diaphragm, and no acute pulmonary process (Figure 3). Laboratory tests presented hypoxemia (arterial partial pressure of oxygen, 73%), a high percentage of peripheral neutrophil (84.7%), increased erythrocyte sedimentation rate (23 mm/h), and hypoxemia (oxygen pressure of 73.4 mmHg). Moreover, cardiac necrosis markers, such as myocardial troponin T (TnT, 1,583 pg/ml; normal reference range, 0–14. pg/ml), creatine kinase (CK, 1,739.5u/L), and creatine kinase isoenzyme-MB (CK-MB, 221.2 u/L) were significantly elevated, with an increased level of myoglobin (2,171.9 ug/ml) and normal level of N-terminal pro-brain natriuretic peptide (NT-proBNP, 81 pg/ml). Considering the symptoms of dyspnea and chest distress and elevation of cardiac necrosis markers, he was preliminarily diagnosed with acute non-ST segment-elevated myocardial infarction and sent to the ward of the department of cardiovascular disease for further treatment.

**Figure 4:**
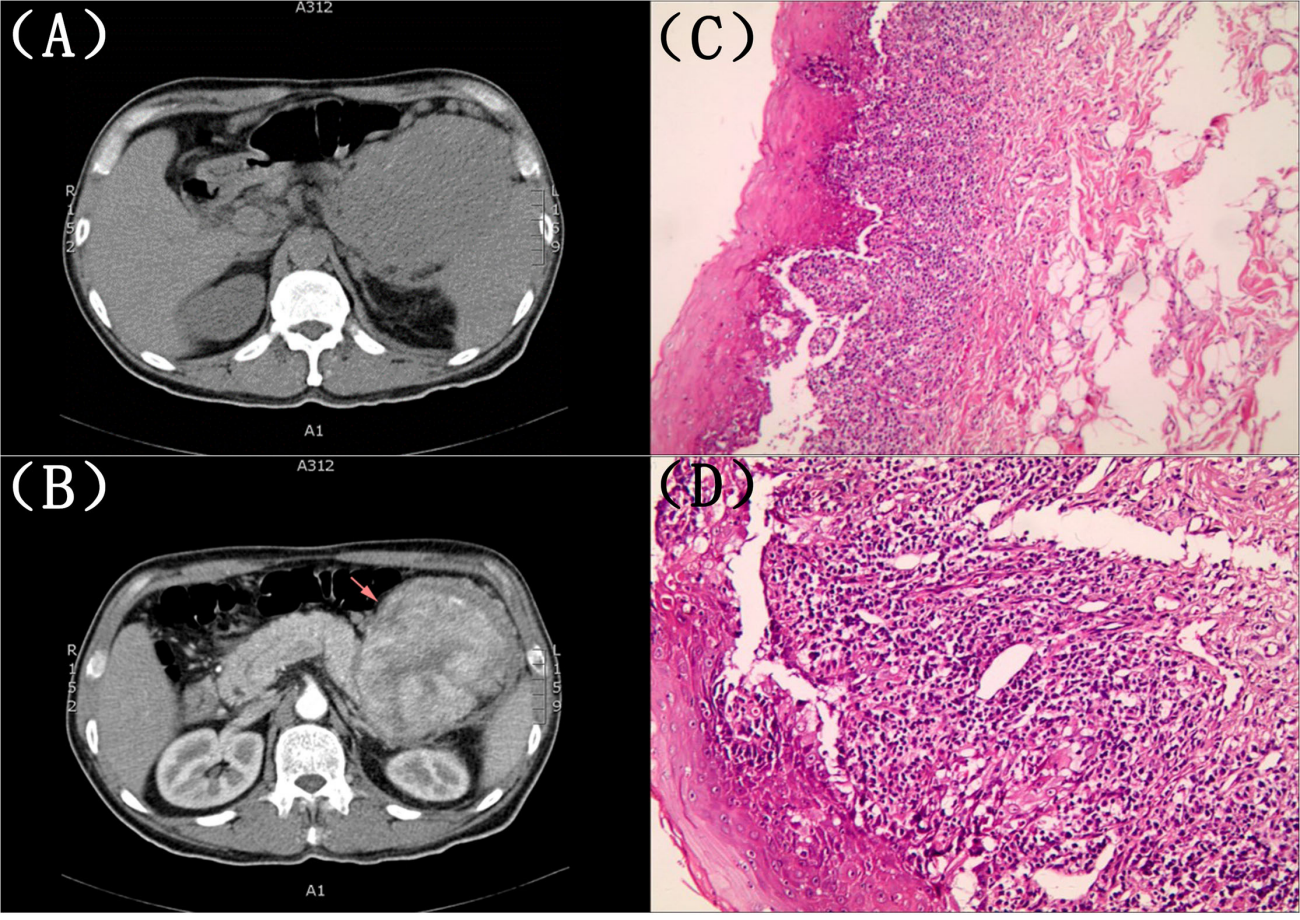
**(A,B)** Computed tomography (CT) scan of the thorax and abdomen. A well-defined tumor between the spleen and stomach, measuring 117 × 108 × 91 mm, accompanied by an abundant blood supply and enlarged retroperitoneal lymph nodes. **(C,D)** The histopathological evaluation of the skin lesions. Suprabasilaracantholysis, keratinocyte necrosis, basal layer damage, and inflammatory cell infiltration in the superficial dermis, including lymphocytes and plasma cells.

The second physical examination revealed some neglected lesions, including nipple scales, genital ulcers, and nail lesions on fingers and toes (Figures 1B–D). A firm mass was found in the left quadrant, which was consistent with the elevation of the dexter diaphragm by the chest X-ray. Electrocardiogram for the second time remained no distinctly dynamic changes (Figure 2B). He was given aspirin, clopidogrel, atorvastatin, nitrates, low molecular heparin, diuretics, and thalidomide after being admitted to the ward. Transthoracic echocardiography revealed the normal size of ventricles and atriums, left ventricle systolic function of 62%, and impaired left ventricular diastolic function (E < A). During his hospitalization, except for a persistent increase of myocardial enzyme (CK-MB: from 172.5 u/L on Day 1 to 228.1 u/L on Day 11 after admission; CK: from 1235.3 u/L on Day 1 to 1081.6 u/L on Day 11 after admission; MB: from 2171.9 to 2368 ug/L) and TnT (from 1,119 pg/ml on Day 1 to 2,295 pg/ml on Day 11 after admission), other laboratory tests, including serum sodium, potassium, blood urea nitrogen, creatinine, and liver function tests, were within normal limits. The serum markers for rheumatic diseases, human immunodeficiency virus, and syphilis infection were also negative. Subsequent computed tomography (CT) scan of the thorax and abdomen demonstrated a well-defined tumor between the spleen and stomach, measuring 117 × 108 × 91 mm, accompanied by the abundant blood supply and enlarged retroperitoneal lymph nodes (Figures 4A,B). No metastasis and pulmonary disease were identified. After antiplatelet, nitrate, and diuretic therapies for 2weeks, the patient still complained of chronic orthopnea and dysphagia caused by severe mouth ulcers and progressive bullae in the mucosa. Taking the persistent raise of CK, CK-MB, and TnT into account, an absolute situation of myocardial damage was considered. However, he was unable to tolerate coronary angiography and cardiac magnetic resonance imaging due to persistent orthopnea.

A biopsy of his lower lip was conducted due to the clinical suspicion of PNP. The histopathological evaluation of the skin lesions revealed suprabasal acantholysis, keratinocyte necrosis, basal layer damage, and inflammatory cell infiltration in the superficial dermis, including lymphocytes and plasma cells, which were compatible with PNP (Figures 4C,D). The direct immunofluorescence (DIF) showed negativity for intercellular deposits of immunoglobulin (Ig) G, IgA, IgM, and Complement 3. Based on the clinical manifestations and pathological findings, PNP was highly suspected after multidisciplinary discussions. He refused the recommendation of surgery therapy or hormonotherapy, autoantibody testing, and left our hospital due to financial issues. The patient was followed up by telephone 3 months later with no improvement of symptoms and was still reluctant to accept surgery.

## Discussion

The PNP is a life-threatening neoplasia-associated autoimmune disease driven by autoantibodies against a complex of desmosomal proteins (8) in multi-organ, and the mortality rate was up to 90%. Patients with PNP frequently have a concurrent malignant or benign neoplasm, mainly including non-Hodgkin's lymphoma, chronic lymphocytic leukemia, and Castleman disease (9). There were also carcinomas that derived from epithelial cells, malignant melanomas, and sarcomas of mesenchymal origin also existed (10). It has a bad prognosis and high mortality due to the production of autoantibodies against internal malignancies and the infiltration of those antibodies into organs other than the skin. Therefore, Nguyen et al. (11) proposed the term “paraneoplastic autoimmune multi-organ syndrome,” instead of PNP, which emphasized the systematic nature of PNP.

The PNP has distinct forms of presentation. Presently, there is no agreement on PNP diagnostic criteria. According to Anahlt et al. (1), the diagnostic criteria include clinical characteristics, histopathology, immunofluorescence (Direct or Indirect), and immunoprecipitation tests. Clinically, the earliest and most consistent finding is severe, refractory, oral mucosal ulceration in the context of an occult/confirmed neoplasm. Histologically, suprabasal acantholysis and keratinocyte necrosis are certain features that are highly specific for PNP. Immunologically, direct immunofluorescence (DIF) indicated interepithelial cell deposition of IgG and C3 with or without linear deposition at the basement membrane zone. Circulating autoantibodies against can be revealed by indirect immunofluorescence (IIF) assays of murine tissue. Immunoprecipitation is characterized by the production of autoantibodies against various target antigens, mainly plakin family proteins (2, 12), including antibodies against the envoplakin, periplakin, desmoplakins I and II, plectin, microtubule-actin cross-linking factor 1, and pemphigus vulgaris antigen (13, 14). In particular, envoplakin and periplakin are the foundation of the primary antigenic proteins in PNP. These proteins act as epidermal cell adhesion molecules and anchor intermediate filaments to desmosomes (15). Furthermore, in addition to humoral responses, the pathophysiology of PNP includes cellular autoimmune responses mediated by CD8+ cytotoxic T lymphocytes, CD56+ natural killer cells, and macrophages (16). More recently, Mimouni et al. (17) modified the original standard of Anahlt et al. In the new classification that DIF was an unnecessary criterion for the diagnosis of PNP, because of its low sensitivity (18). Moreover, he emphasized that IIF labeling of the rat bladder and immunoblotting to identify envoplakin and/or periplakin were necessary for the diagnosis of PNP.

As for our patient, he was misdiagnosed by different hospitals due to significant oral mucosal erosion and was initially treated with metronidazole and cephalosporins with no apparent improvement. Furthermore, after consulting with gastrointestinal surgeons and imaging specialists, our patient's tumor site between the spleen and stomach was considered a malignant mesenchymal neoplasm. Gastrointestinal stromal tumors are the most prevalent kind of gastrointestinal tract and occur anywhere in the gastrointestinal tract (19). There are some typical imaging characteristics: (i) the majority of them are solitary, well-defined round or round-like tumors; (ii) the larger the tumor, the more malignant the tumor is; and (iii) abundant blood supply, followed by enhancement of the parenchyma, which typically displayed apparent enhancement and significant hypo-attenuating necrotic components. The pathological biopsy is the gold standard for diagnosing malignant mesenchymal neoplasm; however, our patient was unable to endure it, owing to persistent orthopnea; therefore, no definitive diagnosis can be made.

It was not until the computed tomography and biopsy were performed that this patient was highly suspected of PNP for his typically clinical manifestations and histological changes. One of the atypical points of this case was the negative result in DIF. However, a positive DIF is not mandatory for diagnosing PNP; it might be negative in certain patients with extensive epidermal destruction (20, 21). This patient occurred to be in this scenario, and his lip ulcerated badly. Furthermore, PNP is also an autoimmune disease that involves both cell-mediated and humoral immunity (22). Lim et al. (23) described a cell-mediated immune response-based PNP that was DIF negative. However, we still think that performing at least one of the following assays to reach the diagnosis of PNP including IIF on rat bladder or immunoblotting to identify envoplakin and/or periplakin.

Although persistent elevation of cardiac necrosis markers was uncommon in previously reported patients with PNP, the increase in cardiac necrosis markers in our case was inconsistent with the typically dynamic changes of acute myocardial infarction, even though coronary angiography was not completed due to orthopnea. Therefore, the etiology of the persistent increase in myocardial necrosis markers may be more complex than a single incident of acute myocardial infarction. Furthermore, the question in this patient was whether the higher indicators of myocardial damage were produced by myocarditis.

Myocarditis is an inflammatory disease of the heart that may occur as a result of infection, exposure to toxic substances, and activation of the immune system (24). Patients with acute myocarditis (AM) (the clinical onset usually <1 month) usually have chest pain as their first symptom (85–95% of cases) (25, 26). Furthermore, fever (about 65%), gastrointestinal disorders, and respiratory infections were also common (ranging from 18 to 80%), and ECG ST-segment elevation in 62.3% of cases. Laboratory tests showed elevated C-reactive protein (80–95%) (27). Endomyocardial biopsy was the gold standard for the definitive diagnosis of myocarditis, but it was usually difficult to complete. For our patient, on the one hand, the clinical symptoms were not consistent with acute myocarditis. On the other hand, the routine laboratory tests revealed that C-reactive protein was 6.30 mg/L (normal reference range, 0–8 pg/ml); respiratory and gastrointestinal viral tests were negative, which was contrary to the laboratory findings of myocarditis. However, the patient refused to have an endomyocardial biopsy, which was pitiful. To summarize, it did indicate PNP-associated cardiac damage in this patient, with the normal systolic function of the left ventricle.

Skin and heart must restrain mechanical forces and need to equip with stress resistance and mechanical flexibility. The intercellular-connecting structures, including the gap, adherens junctions, and desmosomes area composita, display remarkable ultrastructural similarities in both the skin and heart (28). On the one hand, the area composita is a mixed type, adhering junction with high cardiac specificity, connecting cardiac myocytes, and transmitting force during contraction of the mammalian cardiac-intercalated disks (29). Patients with PNP can produce auto-antibodies to the area composita of the heart, such as anti-desmoplakins I and II (3). Desmosomal mutations may play a critical role in PNP cardiac involvement, as previously observed in individuals with cardiocutaneous syndromes (30). On the other hand, other factors may also be involved in troponin elevation: (i) intravascular ultrasonography-verified tumor cells, invading the coronary artery wall and damaging the endothelium to cause myocardial infarction in a prior case report (31). Our patient was unable to tolerate the coronary artery examination and cardiac magnetic resonance imaging due to the incapacity to lie down;(ii) cardiac injury produced by tumor cells infiltrating and pro-inflammatory substances from tumor cells (32); (iii) myocardial oxygen supply imbalance caused by mucosal damage from PNP-related bronchitis.

The discrepancy between orthopnea and the normal level of NT-proBNP and a poor therapeutic response to the treatment of anti-heart failure suggested that there were other causes of dyspnea rather than heart failure, such as pulmonary involvement in PNP. The symptom of progressive dyspnea could be caused by chronic obstructive pulmonary disease or pulmonary vascular disease. The deposition of autoantibodies on the bronchial epithelium would induce bronchiolitis obliterans syndrome presenting as hypoxemia, which is a frequent cause of death (7). Moreover, Wang et al. (33) reported that almost 35% of patients with PNP could be diagnosed with myasthenia gravis, an autoimmune neuromuscular junction disorder nearly accompanied by thymoma. Anti-AChR and anti-AChE, the specific antibodies for myasthenia gravis, are prominent in patients with PNP, especially those with dyspnoea. As for this patient, continuously elevated myohemoglobin also indicated the possibility that skeletal muscle was implicated in PNP. Hence, orthopnoea might be caused by the following reasons: (i) cardiac involvement of PNP; (ii) immune attack toward mucosal epithelium of respiratory tract as a result of the preponderance of desmoplakins; (iii) the weakness of respiratory muscle and diaphragm; and (iiii) the constriction from abdomen due to the huge tumor.

Currently, there is no standard therapy for myocardial damage-associated PNP due to the rarity of this complication. Removal of concomitant neoplasm is considered to be the most effective treatment for patients with PNP. The lesions in the skin and mucosa would disappear after the removal of the concomitant tumor and present once again when the tumor recurs (7).

In conclusion, myocardial damage in our case may be due to the presence of similar autoantibodies against desmosomes, and, thus, apoptosis or necrosis of cardiomyocytes would happen eventually in the end stage of PNP. In summary, we believe that the possibility of combined myocardial damage should be considered in patients with PNP when persistent cardiac necrosis markers are elevated.

## Data Availability Statement

The raw data supporting the conclusions of this article will be made available by the authors, without undue reservation.

## Ethics Statement

Written informed consent was obtained from the individual(s) for the publication of any potentially identifiable images or data included in this article.

## Author Contributions

XD and MZ wrote the report, performed the research, and took pictures. SZ and FT wrote a part of the report. LL and TW diagnosed and treated the patient. All authors read and approved the final version of the manuscript.

## Funding

This work was supported by the National Natural Science Foundation of China projects (Grant Nos. 81270956, 81470577, 202203012938, and kq2202393).

## Conflict of Interest

The authors declare that the research was conducted in the absence of any commercial or financial relationships that could be construed as a potential conflict of interest.

## Publisher's Note

All claims expressed in this article are solely those of the authors and do not necessarily represent those of their affiliated organizations, or those of the publisher, the editors and the reviewers. Any product that may be evaluated in this article, or claim that may be made by its manufacturer, is not guaranteed or endorsed by the publisher.
